# Ionic liquid phase microextraction combined with fluorescence spectrometry for preconcentration and quantitation of carvedilol in pharmaceutical preparations and biological media

**DOI:** 10.1186/s40199-015-0113-x

**Published:** 2015-04-30

**Authors:** Mohsen Zeeb, Behrooz Mirza

**Affiliations:** Department of Applied Chemistry, Faculty of science, Islamic Azad University, South Tehran Branch, Tehran, Iran

**Keywords:** Carvedilol, Hydrophobic ionic liquid, Spectrofluorimetry, Real samples

## Abstract

**Background:**

Carvedilol belongs to a group of medicines termed non-selective beta-adrenergic blocking agents. In the presented approach, a practical and environmentally friendly microextraction method based on the application of ionic liquids (ILs) was followed by fluorescence spectrometry for trace determination of carvedilol in pharmaceutical and biological media.

**Methods:**

A rapid and simple ionic liquid phase microextraction was utilized for preconcentration and extraction of carvedilol. A hydrophobic ionic liquid (IL) was applied as a microextraction solvent. In order to disperse the IL through the aqueous media and extract the analyte of interest, IL was injected into the sample solution and a proper temperature was applied and then for aggregating the IL-phase, the sample was cooled in an ice water-bath. The aqueous media was centrifuged and IL-phase collected at the bottom of the test tube was introduced to the micro-cell of spectrofluorimeter, in order to determine the concentration of the enriched analyte.

**Results:**

Main parameters affecting the accuracy and precision of the proposed approach were investigated and optimized values were obtained. A linear response range of 10–250 μg l^−1^ and a limit of detection (LOD) of 1.7 μg l^−1^ were obtained.

**Conclusion:**

Finally, the presented method was utilized for trace determination of carvedilol in commercial pharmaceutical preparations and biological media.

## Background

**Figure 1:**
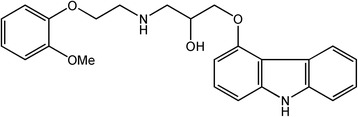
Structure of carvedilol.

Carvedilol belongs to a group of medicines termed non-selective beta-adrenergic blocking agents (Figure 1). This drug is useful in treatment of congestive heart failure. In addition, carvedilol is applied to treat high blood pressure (hypertension) and for prevention of heart attacks [1,2].

In order to assay the presence of carvedilol in pharmaceutical and biological samples, some analytical approaches including chromatography [3-6], spectrophotometery [7], electrochemistry [8,9] and fluorimetry [10] have been developed. These methods suffer form some limitations including poor sensitivity, high cost of analysis, unsuitable selectivity and high time of analysis. One of the best choices for overcoming the mentioned problems is the combination of a practical sample enrichment method with analytical instruments.

In recent years, analytical chemists have developed some practical liquid phase microextraction methods and among these sample pretreatment methods, dispersive liquid-liquid microextraction (DLLME) has received much attention [11,12]. Unfortunately, one of the most important disadvantages of these microextraction methods is the usage of toxic solvents as the extraction solvent such as CHCl_3_, CCl_4_ and etc. In order to remove these toxic materials from microextraction procedures, ionic liquids (ILs) are the best choice. ILs offer many advantages such as low vapor pressure, tunable solubility, desire thermal stability and etc. [13].

In recent years, some microextraction methods based on the application of ILs such as ionic liquid-based dispersive liquid-liquid microextraction (IL-DLLME) [14-16], ionic liquid cold-induced aggregation dispersive liquid-liquid microextraction (IL-CIA-DLLME) [17-19], ionic Liquid-based ultrasound-assisted in situ solvent formation microextraction [20], temperature-controlled ionic liquid dispersive liquid phase microextraction (TCIL-DLPME) [21], etc. have been introduced.

Solubility of ILs depends on the aqueous media temperature; hence it is possible to control the solubility of ILs by changing the temperature. In the presented ionic liquid phase microextraction, in order to disperse the IL-phase into the sample solution and increase the extraction recovery, a high temperature was applied. For collecting the IL-phase, sample solution was cooled and centrifuged.

Our previous studies revealed that the solubility of ILs depends on ionic strength of aqueous media, which has a negative influence on reproducibility and accuracy [18,19]. For solving this problem, a common ion of IL was introduced to the aqueous media. As a result, the solubility of IL phase was not affected by variations of ionic strength, and reproducible volume of enriched phase was obtained.

Some analytical instrument such as spectrofluorimetry offer many advantages such as proper sensitivity, selectivity, cost of analysis, speed of quantitative measurements and etc. In addition, by coupling a microextraction method with fluorescence spectrometry and due to the proper selectivity of this analytical technique, it is avoided the need of employing a high performance separation instrumental for pretreatment of biological samples prior to measurement.

As a part of our continuing efforts for quantitation of drugs using combination of new and benign sample enrichment methods with inexpensive, selective and sensitive analytical instrument [18,21], herein, for the first time a practical and environmentally friendly microextraction method based on the application of ILs was followed with spectrofluorimetry for trace determination of carvedilol in real samples. All variable were evaluated in details and optimized values were obtained.

## Material and methods

### Instrumentation

Detection of fluorescence signals were performed using a Perkin-Elmer LS 50 spectrofluorimeter. This instrument was equipped with xenon discharge lamp, and quartz micro-cell with a volume of 100 μl. Excitation and emission slits were fixed at 15 nm. In order to perform microextraction and optimization steps, a centrifuge from Hettich (Tuttlingen, Germany), a pH-meter, an adjustable sampler (10–100 μL) and a 1 ml syringe was prepared.

### Reagents and materials

Analytical-reagent grade of 1-Hexyl-3-methylimidazolium hexafluorophosphate [Hmim][PF_6_], acetone, acetonitrile, methanol, ethanol, HCl, NaOH and sodium hexafluorophosphate (NaPF_6_) were obtained from Merck (Darmstadt, Germany). A working solution of NaPF_6_ (250 mg ml^-1^) was prepared. For preparing stock solution of carvedilol (1000 mg l^−1^) (Fluka, Switzerland), proper amount of this drug was dissolved in methanol and diluted with ultra pure water. Standard solutions were prepared by dilution of the stock solution with ultra pure water. Tablets containing 12.5 mg and 25 mg carvedilol were purchased from a local pharmacy.

### Sample pretreatment procedure

**Figure 2:**
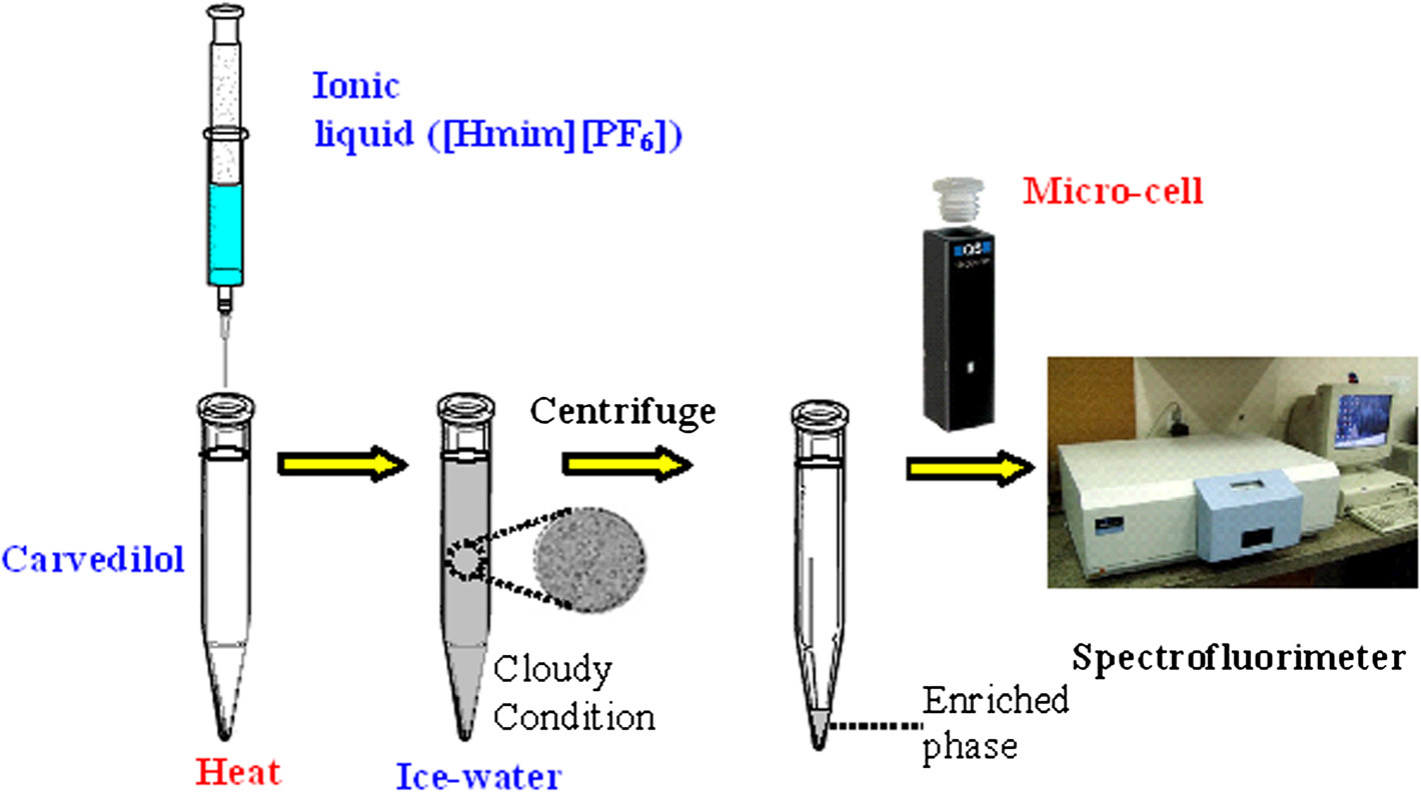
Schematic diagram of the proposed method.

In this sample pretreatment method, ten milliliters of sample solution (10–250 μg l^−1^ of carvedilol) was transferred to a centrifuge tube. The pH of the solution was adjusted at 9. Afterwards, 60 mg of 1-Hexyl-3-methylimidazolium hexafluorophosphate [Hmim][PF_6_] ionic liquid and 0.7 ml of hexafluorophosphate (NaPF_6_) (250 mg ml^−1^) was injected into the aqueous sample solution. After mixing the extractor with sample solution, the resultant solution was transferred into a hot water batch equipped with a thermostat. The temperature of the water batch was fixed at 50°C for 4 min. Under driving the temperature, IL-phase was dissolved and dispersed through the aqueous media. In order to aggregate the IL-phase, the sample was cooled at 0°C for 7 min. In order to collect the enriched phase, sample solution was centrifuged (6 min, 4000 r.p.m). After removing the aqueous media, the enriched phase was diluted with ethanol to 200 μl and transferred into the micro-cell of the spectrofluorimeter. Finally, quantitation of carvedilol was performed. Schematic diagram of the designed method is shown in Figure 2.

### Preparation of pharmaceutical preparations, human urine and human plasma

To obtain pharmaceutical solutions for quantification, eight carvedilol tablets containing 12.5 or 25 mg drug were powdered, mixed and weighted. Required amount of the resultant material containing 10 mg carvedilol was dissolved in methanol with signification. After filtration, the solution was transferred into a 100 ml volumetric vessel and diluted with ultra pure water. In order to set the concentration of carvedilol within the linear response range, further dilution was performed.

For preparing human plasma samples, different concentrations of carvedilol were added to one milliliter of human plasma. After this step, the real sample was deproteinized using 5 ml of acetonitrile. After centrifugation (12 min, 4000 r.p.m), 2.0 ml of the upper phase (clear condition) was diluted with ultra pure water and 10.0 ml of the obtained sample was utilized for quantitation.

In order to prepare human urine samples, ten milliliters of urine were centrifuged (5 min, 4000 r.p.m). Then, 2.0 ml of the upper clear phase was placed in centrifuge test tube and different amount of carvedilol was added to this and diluted to 10.0 ml. Finally, the defined quantitation procedure was performed.

## Results and discussion

In recent work, a simple and benign sample pretreatment method based on the application of ILs was combined with fluorescence spectrometry for enrichment and determination carvedilol in real samples. Main parameters affecting the accuracy and precision of the proposed approach were investigated and optimized values were obtained.

### Fluorescence spectra properties and linear dynamic range

**Figure 3:**
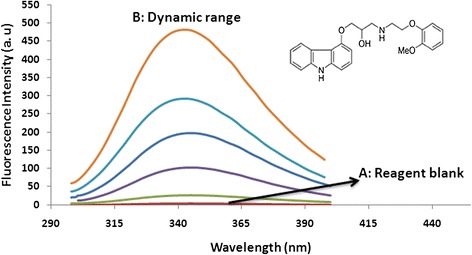
Fluorescence spectra of reagent blank and carvedilol. (A) Fluorescence spectrum of reagent blank after applying microextraction procedure. (B) Fluorescence spectra of carvedilol within linear dynamic range (10, 50, 100, 150, 250 μg l^−1^) after applying microextraction procedure. Applied parameters: sample volume 10 ml; IL 60 mg; NaPF_6_ 175 mg; pH 9; temperature 50°C; λ_ex_ 285 ± 5 nm; λ_em_ 345 ± 5 nm.

Native Fluorescence intensities of molecules with π-electron and cyclic structure are relatively high. As a result, measurement of fluorescence intensity provides a practical tool for sensitive quantitative analysis. After applying the designed microextraction procedure, fluorescence spectra of carvedilol (100 μg l^−1^) was recorded (Figure 3). In this study, emission peaks were recorded at 345 ± 5 nm (excitation wave length was fixed at 285 ± 5 nm).

In order to evaluate the spectra properties of reagent blank, sample pretreatment method was performed without analyte of interest and the fluorescence spectra were recorded at 345 ± 5 nm. No main measurable influence of reagent blank on the quantitative analysis of carvedilol was observed. As a result, these excitation and emission wavelengths were selected for further quantitation of carvedilol.

### Kind of ionic liquid

Based on the results obtained in our previous studies [18,19], three factors must be considered, in order to select a proper IL: (a) the density of IL as the extraction solvent must be higher than aqueous media, (b) IL must illustrate a desire hydrophobicity, (c) IL must be liquid and (d) these ionic material must be inexpensive. ILs with imidazolium scaffold which contain Cl^−^, BF_4_^−^ and CF_3_SO_3_^−^ show hydrophilic properties and those contain PF_6_^−^ and (CF_3_SO_2_)_2_ N^−^ show hydrophobic properties.

According to these factors, [Hmim][PF_6_] was used as an optimum microextraction solvent in all tests.

### Optimization of diluting solvent

The viscosity of ionic liquids is relatively high; hence their direct transfer into the micro-cell of spectrofluorimeter for analyzing carvedilol is difficult. As a result, enriched-phase was conditioned and diluted. For this goal, some conditioner solvents such as methanol, ethanol, acetonitrile and acetone were evaluated as the diluting solvent. The obtained data showed that reproducible and sensitive signals were obtained in using ethanol as a conditioner agent. Due to the better data stability and ethanol environmental safety (less toxicity), this organic solvent was preferred and used in all experiments.

### Optimization of IL amount

**Figure 4:**
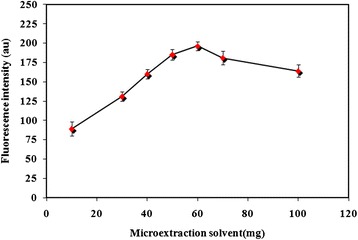
Effect of IL as the microextraction phase. Applied parameters: Carvedilol concentration 100 μg l^−1^; sample volume 10 ml; NaPF_6_ 175 mg; pH 9; temperature 50°C; λ_ex_ 285 ± 5 nm; λ_em_ 345 ± 5 nm. Indicated analytical signals are the average of three independent measurements and error bars correspond to their standard deviations.

As it was mentioned, in this microextraction procedure, IL was applied as the microextraction phase. In this kind of sample pretreatment method, one of the major parameters affecting the performance is the amount of IL. This parameter has a significant effect on the reproducibility and sensitivity. In order to optimize the amount of extraction solvent, this parameter was tested within the range of 10–100 mg (Figure 4). Stable and sensitive fluorescence signals were obtained at 60 mg and this value was used for the rest of the work.

### Optimization of PF_6_^−^ amount and ionic strength

**Figure 5:**
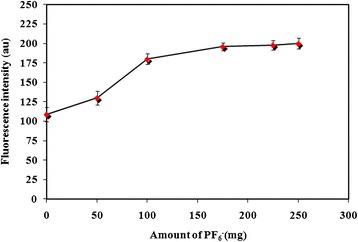
Effect of PF_6_^−^ Applied parameters: Carvedilol concentration 100 μg l^−1^; Sample volume 10 ml; IL 60 mg; pH 9; temperature 50°C; λ_ex_ 285 ± 5 nm; λ_em_ 345 ± 5 nm. Indicated analytical signals are the average of three independent measurements and error bars correspond to their standard deviations.

As it was demonstrated in our previous works [18,19], dissolving a common ion of IL like PF_6_^−^, significantly reduce the solubility of IL. This act improves the extraction performance of carvedilol and provides better analytical sensitivity. Effect of this parameter was examined in the range of 0–250 mg (see Figure 5). A value of 175 mg was selected as an optimum value, in order to obtain proper signal stability and reproducibility.

One of the most important parameters which affects on the extraction performance is ionic strength of the aqueous media. An increase in ionic strength causes a considerable increase in solubility of IL. As a result, the volume of the settled phase depends on the salt content of the sample solution. This phenomenon has a negative influence on the stability of analytical data. Fortunately, presence of PF_6_^−^ (as a common ion) solves this problem and fixes the volume of the enrich phase. The effect of ionic strength was studied within the range of 0–40% (w/v) using NaNO_3_ as an electrolyte. In the studied range, no significant influence on fluorescence signal was observed.

### Optimization of pH

**Figure 6:**
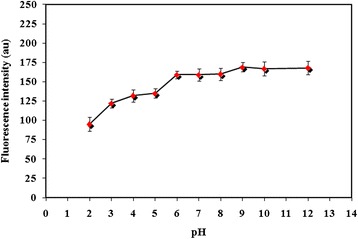
Effect of pH. Applied parameters: Carvedilol concentration 100 μg l^-1^; sample volume 10 ml; IL 60 mg; NaPF_6_ 175 mg; temperature 50°C; λ_ex_ 285 ± 5 nm; λ_em_ 345 ± 5 nm. Indicated analytical signals are the average of three independent measurements and error bars correspond to their standard deviations.

In the case of microextraction of molecules like carvedilol, which have ionizable property, pH of the aqueous media reveals a significant role. In order to obtain the highest extraction efficiency, the uncharged condition of carvedilol must be prevalent (pK_a_ value of carvedilol is 7.97) [22]. The effect of sample pH on the analytical sensitivity and reproducibility was tested within the range of 2–12 (Figure 6). In the recent experiments, HCl and NaOH were used for adjusting the pH. Based on the results obtained in this study, in order to obtain a compromise between sensitivity and reproducibility, pH 9 was selected for further experiments.

### Influence of temperature

In this microextraction procedure, IL-phase is dispersed into the aqueous media under increasing the temperature. The effect of this parameter was evaluated in the range of 25–80°C. Finally, a temperature of 50°C was used as an optimum value. In order to collect the IL-phase after extraction, the sample solution must be cooled. For the recent goal, the aqueous media was placed in ice-water bath and kept at 0°C for 7 min.

### Interference study

For studying the possible interferences coming form other compounds, which exist in real samples, some ions and compounds were subjected to the recent combined methodology. In this investigation, the effect of 100-fold of K^+^, Na^+^, Mg^+^, F^−^, Cl^−^_,_ NO_3_^−^, SO_4_^2−^, glucose, urea, lactic acid, sucrose, ascorbic acid and fructose as the interfering or quenching agents on the determination of carvedilol (100 μg L^−1^) was evaluated. No change in signals over than 4.5% was observed.

### Analytical figures of merits

Linear analytical response range was defined by analyzing standard solutions of carvedilol. The obtained results revealed that analytical responses are linear from 10 to 250 μg l^−1^. Other analytical figures of merits obtained by the ionic liquid phase microextraction-spectrofluorimetry are shown in Table 1. Limit of detection (LOD) was determined using a conventional equation, LOD = ks_bl_/m. This equation is resulted from the equation showed below:

$$ \begin{array}{l}{S}_m={S}_{bl}+{ks}_{bl}\\ {}{S}_m=m{c}_m+{S}_{bl}\\ {}{c}_m=\frac{S_m-{S}_{bl}}{m}=\frac{ks_{bl}}{m}\end{array} $$

S_m_, S_bl_, s_bl_, K, m and C_m_ show the minimum distinguishable analytical signal, average of blank analytical signal, blank standard deviation, constant value equal with 3 (confidence level of 95%), calibration graph slope and detection limit, respectively. Using this way, a value of 1.7 μg l^−1^ carvedilol was achieved. In order to determine the relative standard deviation (RSD), four 100 μg l^−1^ of carvedilol was subjected to the designed methodology and finally a value of 3.8% was obtained.

**Table 1:** **Analytical characteristics of the presented work**

**Analytical factor**	**Values**
Linear analytical response range (μg l^−1^)	10-250
Correlation coefficient (R^2^)	0.9980
LOD^a^ (μg l^−1^)	1.7
RSD^b^ (%) (n = 4) (C_carvedilol_ = 100 μg l^−1^)	3.8
PF^c^	50
Sample volume (mL)	10

^a^Limit of detection.

^b^Relative standard deviation.

^c^The ratio of diluted settled phase volume to aqueous volume gives the preconcentration factor (PF).

### Comparison with reported methods

In order to show the analytical advantages of the proposed method for the quantitation of carvedilol, some details were compared with reported methods in literature, and these results are shown in Table 2. As it can be seen, considerable LOD and relatively wide dynamic range were obtained. In addition, in most of the reported methods, tedious sample pretreatment procedures, toxic solvents and expensive analytical instrument have been used for quantification. In contrast, in the proposed method, a rapid, benign and simple ionic liquid phase microextraction was utilized for preconcentration and extraction of carvedilol. No hazardous material was used in this sample pre-treatment method. In addition, an inexpensive and sensitive analytical instrument was applied for quantitation.

**Table 2:** **Comparison of the proposed methodology with reported methods**

**Method**	**Sample**	**LOD (μg l** ^**−1**^ **)**	**LR (μg l** ^**−1**^ **)**	**Reference**
DLLME-HPLC^a^	Human Urine, Human Plasma	4, 14	50-750, 20-1000	[6]
SPE-CE^b^	Human Urine	50	50-500	[23]
Synchronous fluorimetry	Pharmaceutical preparations	1	5-100	[24]
LLE-HPLC^c^	Human serum	2.5	5-500	[25]
Ionic liquid phase microextraction-spectrofluorimetry	Human Urine, Human Plasma, Pharmaceutical preparations	1.7	10-250	This work

^a^Dispersive liquid-liquid microextraction.

^b^Solid phase extraction-capillary electrophoresis.

^c^Liquid-liquid extraction.

### Analysis of carvedilol in real samples

In order to demonstrate the analytical application of the presented technique, real samples including human urine and human plasma were spiked with different amounts of carvedilol and analyzed. Results of this investigation are shown in Table 3. At it can be seen, the averages of recoveries are placed in the range of 97.4-106.2% (urine) and 89.2-107.3% (plasma). It can be concluded that in the case of accuracy and reproducibility, satisfactory results were obtained. In the next step, some commercial pharmaceutical formulations involving carvedilol capsules and tablets were subjected to the designed method, in order to determine concentration of carvedilol (Table 4). The results obtained with the present work were compared with a reported method [5]. These data reveal the practical analytical application of the proposed method for analyzing the analyte of interest in pharmaceutical preparations.

**Table 3:** **Results of recoveries of spiked biological samples**

**Sample**	**Carvedilol added (μg l** ^**−1**^ **)**	**Carvedilol found (μg l** ^**−1**^ **)** ^**a**^	**RSD (%)**	**Recovery (%)**
Urine	50	53.2	6.1	106.4
	100	98.3	5.9	98.3
	150	146.1	3.8	97.4
Plasma	50	44.6	5.6	89.2
	100	107.3	7.1	107.3
	150	154.8	4.2	103.2

^a^Average of four independent measurements.

**Table 4:** **Analysis of carvedilol tablets by the present work and the reported method (5)**

**Claimed (mg/tablet)**	**Proposed method (mg)** ^**a**^	**Reported method (mg)** ^**a**^	**Error (%)** ^**b**^	**Error (%)** ^**c**^
12.5	12.3 (±0.5)	12.6 (±0.4)	−1.6	−2.9
25	25.5 (±1.0)	25.7 (±1.1)	+2.0	−0.7

^a^Values in parenthesis give the standard deviation based on four determinations.

^b^Error against the tablet value.

^c^Error against the reported method.

## Conclusion

A rapid, benign and simple ionic liquid phase microextraction was utilized for preconcentration and extraction of carvedilol. The enriched-phase was introduced to spectrofluorimeter for quantitation of carvedilol. No toxic and hazardous material was used in this sample pre-treatment method. In addition, an inexpensive and sensitive analytical instrument was applied for quantitative measurements. Finally, the combined methodology was successfully applied for quantitation of carvedilol in real samples.
